# 4,5-Bis(4-meth­oxy­phen­oxy)phthalonitrile

**DOI:** 10.1107/S1600536810035610

**Published:** 2010-09-11

**Authors:** Lijuan Yu, Xiaole Zhou, Yinghui Yin, Renjie Li, Tianyou Peng

**Affiliations:** a College of Chemistry and Molecular Science, Wuhan University, Wuhan 430072, People’s Republic of China

## Abstract

The title compound, C_22_H_16_N_2_O_4_, was obtained unintentionally as the product of an attempted synthesis of a new phthalocyanine. The dihedral angles formed by the central benzene ring with the aromatic rings of the meth­oxy­phen­oxy groups are 85.39 (5) and 64.19 (5)°.

## Related literature

For background information on phthalcoyanines, see: Hanack & Lang (1994 ▶). For the synthesis of the title compound, see: Li *et al.* (2006 ▶).
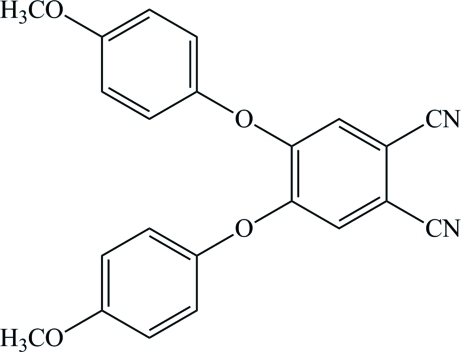


## Experimental

### 

#### Crystal data

C_22_H_16_N_2_O_4_
*M* *_r_* = 372.37
Monoclinic, 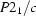

*a* = 13.7614 (2) Å
*b* = 10.4926 (1) Å
*c* = 14.0701 (2) Å
β = 112.551 (1)°
*V* = 1876.28 (4) Å^3^
*Z* = 4
Mo *K*α radiation
μ = 0.09 mm^−1^
*T* = 298 K
0.38 × 0.23 × 0.13 mm

#### Data collection

Bruker APEXII CCD area-detector diffractometer
Absorption correction: multi-scan (*SADABS*; Sheldrick, 2004 ▶) *T*_min_ = 0.966, *T*_max_ = 0.988
24018 measured reflections
3311 independent reflections
2189 reflections with *I* > 2σ(*I*)
*R*_int_ = 0.032

#### Refinement

*R*[*F* ^2^ > 2σ(*F* ^2^)] = 0.038
*wR*(*F* ^2^) = 0.097
*S* = 1.02
3311 reflections
255 parameters
H-atom parameters constrained
Δρ_max_ = 0.13 e Å^−3^
Δρ_min_ = −0.17 e Å^−3^

### 

Data collection: *APEX2* (Bruker, 2004 ▶); cell refinement: *SAINT-Plus* (Bruker, 2001 ▶); data reduction: *SAINT-Plus*; program(s) used to solve structure: *SHELXS97* (Sheldrick, 2008 ▶); program(s) used to refine structure: *SHELXL97* (Sheldrick, 2008 ▶); molecular graphics: *SHELXTL* (Sheldrick, 2008 ▶); software used to prepare material for publication: *SHELXL97*.

## Supplementary Material

Crystal structure: contains datablocks I, global. DOI: 10.1107/S1600536810035610/rz2481sup1.cif

Structure factors: contains datablocks I. DOI: 10.1107/S1600536810035610/rz2481Isup2.hkl

Additional supplementary materials:  crystallographic information; 3D view; checkCIF report

## Figures and Tables

**Fig. 1.**
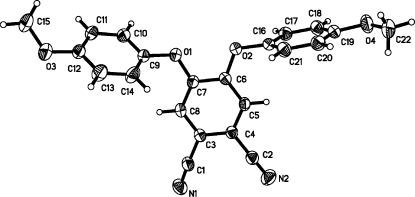
The molecular structure of (I), with atom labels and 30% probability displacement ellipsoids for non-H atoms.

## supplementary crystallographic information

### Comment

In the past few years, phthalocyanines have been extensively studied for their high thermal stability as well as their wide application fields (Hanack & Lang, 1994). As part of our ongoing studies of phthalocyanines (Li *et al.*, 2006), we report herein the synthesis and crystal structure of the title compound.

The molecular structure of the title compound is shown in Fig. 1. The C—N bond lengths within each C≡N group are almost equal, with an average value of 1.141 (2) Å. The C and O atoms of the central 1,2-dioxybenzene group and the cyanide groups are substantially coplanar [maximum deviation 0.029 (2) Å for atom N2] and form dihedral angles of 85.39 (5) and 64.19 (5)° with the C16—C21 and C9—C14 benzene rings. The crystal packing is stabilized only by van der Waals interactions.

### Experimental

The title compound was prepared according to the literarure method (Li *et al.*, 2006), using vapour diffusion of ethanol into a toluene solution of the title compound at room temperature. Analysis calculated (%): C 70.96, N 7.52, H 4.33; found(%): C 70.51, N 7.21, H 4.19. ^1^H NMR (CDCl_3_, δ, p.p.m.): 7.95 (s, 2H), 7.36 (d, 4H), 6.92 (m, 4H), 3.90 (s, 6H).

### Refinement

All H atoms were positioned geometrically and constrained to ride on their parent atoms, with C—H = 0.93–0.96 Å, and with *U*_iso_(H) = 1.2 *U*_eq_(C) or 1.5*U*_eq_(C) for methyl H atoms.

### Crystal data

**Table:**

C_22_H_16_N_2_O_4_	*F*(000) = 776
*M**_r_* = 372.37	*D*_x_ = 1.318 Mg m^−^^3^
Monoclinic, *P*2_1_/*c*	Mo *K*α radiation, λ = 0.71073 Å
Hall symbol: -P 2ybc	Cell parameters from 5549 reflections
*a* = 13.7614 (2) Å	θ = 2.5–23.5°
*b* = 10.4926 (1) Å	µ = 0.09 mm^−^^1^
*c* = 14.0701 (2) Å	*T* = 298 K
β = 112.551 (1)°	Block, colourless
*V* = 1876.28 (4) Å^3^	0.38 × 0.23 × 0.13 mm
*Z* = 4	

### Data collection

**Table:**

Bruker APEXII CCD area-detector diffractometer	3311 independent reflections
Radiation source: fine-focus sealed tube	2189 reflections with *I* > 2σ(*I*)
graphite	*R*_int_ = 0.032
ω scan	θ_max_ = 25.0°, θ_min_ = 1.6°
Absorption correction: multi-scan (*SADABS*; Sheldrick, 2004)	*h* = −16→16
*T*_min_ = 0.966, *T*_max_ = 0.988	*k* = −11→12
24018 measured reflections	*l* = −16→16

### Refinement

**Table:**

Refinement on *F*^2^	Primary atom site location: structure-invariant direct methods
Least-squares matrix: full	Secondary atom site location: difference Fourier map
*R*[*F*^2^ > 2σ(*F*^2^)] = 0.038	Hydrogen site location: inferred from neighbouring sites
*wR*(*F*^2^) = 0.097	H-atom parameters constrained
*S* = 1.01	*w* = 1/[σ^2^(*F*_o_^2^) + (0.0369*P*)^2^ + 0.4099*P*] where *P* = (*F*_o_^2^ + 2*F*_c_^2^)/3
3311 reflections	(Δ/σ)_max_ < 0.001
255 parameters	Δρ_max_ = 0.13 e Å^−^^3^
0 restraints	Δρ_min_ = −0.17 e Å^−^^3^

### Special details

**Table:**

Geometry. All e.s.d.'s (except the e.s.d. in the dihedral angle between two l.s. planes) are estimated using the full covariance matrix. The cell e.s.d.'s are taken into account individually in the estimation of e.s.d.'s in distances, angles and torsion angles; correlations between e.s.d.'s in cell parameters are only used when they are defined by crystal symmetry. An approximate (isotropic) treatment of cell e.s.d.'s is used for estimating e.s.d.'s involving l.s. planes.
Refinement. Refinement of *F*^2^ against ALL reflections. The weighted *R*-factor *wR* and goodness of fit *S* are based on *F*^2^, conventional *R*-factors *R* are based on *F*, with *F* set to zero for negative *F*^2^. The threshold expression of *F*^2^ > σ(*F*^2^) is used only for calculating *R*-factors(gt) *etc*. and is not relevant to the choice of reflections for refinement. *R*-factors based on *F*^2^ are statistically about twice as large as those based on *F*, and *R*- factors based on ALL data will be even larger.

### Fractional atomic coordinates and isotropic or equivalent isotropic displacement parameters (Å^2^)

**Table:**

	*x*	*y*	*z*	*U*_iso_*/*U*_eq_	
C17	0.07245 (13)	0.02765 (18)	0.29042 (13)	0.0566 (5)	
H17	0.0524	0.1096	0.2653	0.068*	
N2	0.54537 (15)	0.17422 (18)	0.53262 (13)	0.0822 (6)	
N1	0.63571 (15)	0.30662 (18)	0.31740 (13)	0.0820 (5)	
C2	0.49439 (14)	0.16026 (18)	0.44763 (15)	0.0572 (5)	
C1	0.55634 (16)	0.25589 (18)	0.29120 (14)	0.0598 (5)	
O1	0.22453 (9)	0.09523 (14)	0.03832 (9)	0.0684 (4)	
O2	0.16999 (9)	0.01007 (12)	0.18264 (8)	0.0621 (4)	
O3	0.33469 (11)	0.01535 (14)	−0.29526 (10)	0.0796 (4)	
O4	0.02251 (11)	−0.19057 (15)	0.46372 (11)	0.0843 (5)	
C5	0.33212 (13)	0.08186 (16)	0.31600 (12)	0.0496 (4)	
H5	0.3124	0.0507	0.3679	0.060*	
C3	0.45763 (13)	0.19033 (16)	0.26340 (13)	0.0494 (4)	
C7	0.29686 (13)	0.11161 (17)	0.13679 (12)	0.0510 (4)	
C6	0.26639 (13)	0.06676 (16)	0.21448 (12)	0.0485 (4)	
C4	0.42795 (13)	0.14356 (16)	0.34096 (12)	0.0475 (4)	
C8	0.39146 (13)	0.17408 (16)	0.16126 (13)	0.0534 (5)	
H8	0.4109	0.2053	0.1092	0.064*	
C9	0.25752 (13)	0.07923 (17)	−0.04349 (12)	0.0513 (4)	
C11	0.21973 (15)	0.11325 (19)	−0.22164 (13)	0.0611 (5)	
H11	0.1780	0.1501	−0.2841	0.073*	
C12	0.30366 (15)	0.03855 (18)	−0.21509 (13)	0.0561 (5)	
C10	0.19704 (14)	0.13386 (19)	−0.13506 (13)	0.0599 (5)	
H10	0.1404	0.1851	−0.1394	0.072*	
C14	0.34035 (16)	0.0024 (2)	−0.03671 (14)	0.0701 (6)	
H14	0.3807	−0.0364	0.0253	0.084*	
C15	0.25790 (19)	0.0227 (2)	−0.39650 (15)	0.0916 (7)	
H15A	0.1982	−0.0284	−0.4017	0.137*	
H15B	0.2871	−0.0083	−0.4441	0.137*	
H15C	0.2362	0.1097	−0.4125	0.137*	
C13	0.36321 (16)	−0.0166 (2)	−0.12246 (15)	0.0729 (6)	
H13	0.4200	−0.0679	−0.1177	0.087*	
C16	0.13818 (13)	−0.04202 (18)	0.25815 (12)	0.0500 (4)	
C19	0.06522 (13)	−0.14685 (19)	0.39663 (13)	0.0562 (5)	
C18	0.03643 (14)	−0.02478 (19)	0.36030 (14)	0.0611 (5)	
H18	−0.0076	0.0223	0.3832	0.073*	
C20	0.13229 (14)	−0.21514 (19)	0.36496 (14)	0.0605 (5)	
H20	0.1529	−0.2968	0.3903	0.073*	
C21	0.16913 (14)	−0.16199 (19)	0.29513 (14)	0.0610 (5)	
H21	0.2148	−0.2077	0.2735	0.073*	
C22	0.05311 (19)	−0.3141 (2)	0.50731 (17)	0.0959 (8)	
H22A	0.0348	−0.3764	0.4533	0.144*	
H22B	0.0174	−0.3333	0.5523	0.144*	
H22C	0.1278	−0.3156	0.5457	0.144*	

### Atomic displacement parameters (Å^2^)

**Table:**

	*U*^11^	*U*^22^	*U*^33^	*U*^12^	*U*^13^	*U*^23^
C17	0.0538 (11)	0.0585 (12)	0.0579 (11)	0.0016 (9)	0.0218 (9)	0.0032 (9)
N2	0.0906 (13)	0.0919 (14)	0.0548 (11)	−0.0179 (11)	0.0175 (10)	−0.0080 (9)
N1	0.0812 (12)	0.0891 (13)	0.0840 (12)	−0.0312 (11)	0.0409 (10)	−0.0140 (10)
C2	0.0604 (11)	0.0590 (12)	0.0547 (12)	−0.0090 (9)	0.0249 (10)	−0.0040 (9)
C1	0.0674 (13)	0.0617 (13)	0.0582 (12)	−0.0135 (11)	0.0330 (10)	−0.0070 (9)
O1	0.0540 (7)	0.1085 (11)	0.0438 (7)	−0.0043 (7)	0.0200 (6)	0.0038 (7)
O2	0.0499 (7)	0.0901 (10)	0.0466 (7)	−0.0137 (7)	0.0187 (6)	0.0044 (6)
O3	0.0843 (10)	0.1044 (12)	0.0543 (8)	0.0018 (8)	0.0311 (7)	−0.0108 (8)
O4	0.0803 (10)	0.1067 (12)	0.0830 (10)	0.0058 (9)	0.0502 (8)	0.0287 (9)
C5	0.0543 (11)	0.0557 (11)	0.0444 (10)	0.0003 (9)	0.0250 (8)	0.0026 (8)
C3	0.0525 (10)	0.0480 (10)	0.0533 (10)	−0.0039 (8)	0.0263 (9)	−0.0024 (8)
C7	0.0521 (10)	0.0591 (11)	0.0427 (10)	0.0022 (9)	0.0193 (8)	0.0028 (8)
C6	0.0453 (10)	0.0532 (11)	0.0495 (10)	−0.0009 (8)	0.0211 (8)	0.0016 (8)
C4	0.0506 (10)	0.0473 (10)	0.0463 (10)	−0.0005 (8)	0.0205 (8)	−0.0028 (8)
C8	0.0591 (11)	0.0591 (12)	0.0502 (10)	−0.0017 (9)	0.0302 (9)	0.0042 (9)
C9	0.0506 (10)	0.0611 (12)	0.0423 (10)	−0.0062 (9)	0.0179 (8)	0.0023 (8)
C11	0.0629 (12)	0.0727 (13)	0.0433 (10)	−0.0001 (10)	0.0155 (9)	0.0107 (9)
C12	0.0610 (12)	0.0617 (12)	0.0463 (10)	−0.0065 (10)	0.0213 (9)	−0.0047 (9)
C10	0.0532 (11)	0.0723 (13)	0.0523 (11)	0.0070 (10)	0.0180 (9)	0.0086 (10)
C14	0.0786 (14)	0.0786 (15)	0.0464 (11)	0.0182 (11)	0.0166 (10)	0.0123 (10)
C15	0.1065 (18)	0.120 (2)	0.0493 (12)	−0.0121 (15)	0.0310 (12)	−0.0134 (12)
C13	0.0765 (14)	0.0812 (15)	0.0571 (12)	0.0228 (11)	0.0213 (11)	−0.0005 (11)
C16	0.0428 (9)	0.0633 (12)	0.0441 (9)	−0.0084 (9)	0.0166 (8)	0.0005 (9)
C19	0.0463 (10)	0.0733 (14)	0.0511 (10)	−0.0036 (10)	0.0211 (9)	0.0057 (10)
C18	0.0558 (11)	0.0723 (14)	0.0632 (12)	0.0069 (10)	0.0316 (10)	0.0005 (10)
C20	0.0590 (11)	0.0601 (12)	0.0620 (12)	0.0040 (10)	0.0229 (10)	0.0087 (10)
C21	0.0575 (11)	0.0706 (14)	0.0613 (11)	0.0063 (10)	0.0298 (9)	−0.0009 (10)
C22	0.0987 (17)	0.113 (2)	0.0784 (15)	−0.0085 (15)	0.0363 (13)	0.0389 (14)

### Geometric parameters (Å, °)

**Table:**

C17—C16	1.369 (2)	C9—C10	1.363 (2)
C17—C18	1.374 (2)	C9—C14	1.369 (2)
C17—H17	0.9300	C11—C12	1.369 (3)
N2—C2	1.140 (2)	C11—C10	1.386 (2)
N1—C1	1.142 (2)	C11—H11	0.9300
C2—C4	1.438 (2)	C12—C13	1.373 (3)
C1—C3	1.437 (3)	C10—H10	0.9300
O1—C7	1.3719 (19)	C14—C13	1.373 (3)
O1—C9	1.400 (2)	C14—H14	0.9300
O2—C6	1.3633 (19)	C15—H15A	0.9600
O2—C16	1.4057 (19)	C15—H15B	0.9600
O3—C12	1.372 (2)	C15—H15C	0.9600
O3—C15	1.413 (2)	C13—H13	0.9300
O4—C19	1.369 (2)	C16—C21	1.366 (2)
O4—C22	1.426 (3)	C19—C20	1.371 (2)
C5—C6	1.376 (2)	C19—C18	1.380 (3)
C5—C4	1.388 (2)	C18—H18	0.9300
C5—H5	0.9300	C20—C21	1.384 (2)
C3—C8	1.386 (2)	C20—H20	0.9300
C3—C4	1.393 (2)	C21—H21	0.9300
C7—C8	1.378 (2)	C22—H22A	0.9600
C7—C6	1.394 (2)	C22—H22B	0.9600
C8—H8	0.9300	C22—H22C	0.9600
			
C16—C17—C18	119.27 (18)	C9—C10—C11	120.23 (18)
C16—C17—H17	120.4	C9—C10—H10	119.9
C18—C17—H17	120.4	C11—C10—H10	119.9
N2—C2—C4	178.6 (2)	C9—C14—C13	119.32 (17)
N1—C1—C3	177.2 (2)	C9—C14—H14	120.3
C7—O1—C9	120.46 (13)	C13—C14—H14	120.3
C6—O2—C16	117.85 (12)	O3—C15—H15A	109.5
C12—O3—C15	118.08 (16)	O3—C15—H15B	109.5
C19—O4—C22	117.84 (16)	H15A—C15—H15B	109.5
C6—C5—C4	119.94 (15)	O3—C15—H15C	109.5
C6—C5—H5	120.0	H15A—C15—H15C	109.5
C4—C5—H5	120.0	H15B—C15—H15C	109.5
C8—C3—C4	119.71 (16)	C14—C13—C12	121.19 (19)
C8—C3—C1	121.20 (16)	C14—C13—H13	119.4
C4—C3—C1	119.08 (15)	C12—C13—H13	119.4
O1—C7—C8	124.19 (15)	C21—C16—C17	120.99 (16)
O1—C7—C6	115.44 (15)	C21—C16—O2	120.27 (16)
C8—C7—C6	120.27 (15)	C17—C16—O2	118.68 (17)
O2—C6—C5	124.12 (15)	O4—C19—C20	124.73 (18)
O2—C6—C7	115.91 (14)	O4—C19—C18	115.37 (17)
C5—C6—C7	119.96 (16)	C20—C19—C18	119.89 (17)
C5—C4—C3	120.11 (15)	C17—C18—C19	120.37 (17)
C5—C4—C2	118.90 (15)	C17—C18—H18	119.8
C3—C4—C2	120.98 (15)	C19—C18—H18	119.8
C7—C8—C3	119.99 (16)	C19—C20—C21	119.71 (18)
C7—C8—H8	120.0	C19—C20—H20	120.1
C3—C8—H8	120.0	C21—C20—H20	120.1
C10—C9—C14	120.23 (17)	C16—C21—C20	119.74 (17)
C10—C9—O1	116.98 (16)	C16—C21—H21	120.1
C14—C9—O1	122.49 (16)	C20—C21—H21	120.1
C12—C11—C10	119.92 (17)	O4—C22—H22A	109.5
C12—C11—H11	120.0	O4—C22—H22B	109.5
C10—C11—H11	120.0	H22A—C22—H22B	109.5
C11—C12—O3	124.51 (17)	O4—C22—H22C	109.5
C11—C12—C13	119.09 (18)	H22A—C22—H22C	109.5
O3—C12—C13	116.40 (18)	H22B—C22—H22C	109.5
			
C9—O1—C7—C8	33.4 (3)	C15—O3—C12—C11	−26.5 (3)
C9—O1—C7—C6	−150.32 (16)	C15—O3—C12—C13	154.53 (19)
C16—O2—C6—C5	−3.4 (2)	C14—C9—C10—C11	−0.7 (3)
C16—O2—C6—C7	177.01 (15)	O1—C9—C10—C11	−174.62 (16)
C4—C5—C6—O2	−178.51 (15)	C12—C11—C10—C9	−0.5 (3)
C4—C5—C6—C7	1.0 (3)	C10—C9—C14—C13	1.4 (3)
O1—C7—C6—O2	1.4 (2)	O1—C9—C14—C13	174.93 (18)
C8—C7—C6—O2	177.87 (15)	C9—C14—C13—C12	−0.9 (3)
O1—C7—C6—C5	−178.18 (15)	C11—C12—C13—C14	−0.4 (3)
C8—C7—C6—C5	−1.7 (3)	O3—C12—C13—C14	178.69 (18)
C6—C5—C4—C3	0.1 (2)	C18—C17—C16—C21	−0.7 (3)
C6—C5—C4—C2	178.85 (16)	C18—C17—C16—O2	176.69 (15)
C8—C3—C4—C5	−0.5 (2)	C6—O2—C16—C21	−83.9 (2)
C1—C3—C4—C5	178.88 (16)	C6—O2—C16—C17	98.69 (18)
C8—C3—C4—C2	−179.28 (16)	C22—O4—C19—C20	2.7 (3)
C1—C3—C4—C2	0.1 (2)	C22—O4—C19—C18	−177.31 (17)
O1—C7—C8—C3	177.40 (16)	C16—C17—C18—C19	−0.7 (3)
C6—C7—C8—C3	1.3 (3)	O4—C19—C18—C17	−178.31 (16)
C4—C3—C8—C7	−0.2 (3)	C20—C19—C18—C17	1.7 (3)
C1—C3—C8—C7	−179.53 (16)	O4—C19—C20—C21	178.76 (17)
C7—O1—C9—C10	−143.22 (17)	C18—C19—C20—C21	−1.2 (3)
C7—O1—C9—C14	43.1 (2)	C17—C16—C21—C20	1.1 (3)
C10—C11—C12—O3	−177.94 (17)	O2—C16—C21—C20	−176.20 (15)
C10—C11—C12—C13	1.1 (3)	C19—C20—C21—C16	−0.2 (3)
